# Effects of Benzoquinones on Radicles of *Orobanche* and *Phelipanche* Species

**DOI:** 10.3390/plants10040746

**Published:** 2021-04-11

**Authors:** Mónica Fernández-Aparicio, Marco Masi, Alessio Cimmino, Antonio Evidente

**Affiliations:** 1 Institute for Sustainable Agriculture-CSIC, Avda. Menéndez Pidal sn, 14004 Córdoba, Spain; 2 Department of Chemical Sciences, University of Naples Federico II, Complesso Universitario Monte S. Angelo, Via Cintia, 80126 Naples, Italy; marco.masi@unina.it (M.M.); alessio.cimmino@unina.it (A.C.)

**Keywords:** allelopathy, broomrape weeds, growth inhibition, haustorium, 2,6-dimethoxy-*p*-benzoquinone (DMBQ), *p*-benzoquinone (BQ), sustainable crop protection

## Abstract

The holoparasitic broomrape weeds (*Orobanche* and *Phelipanche* species) cause severe yield losses throughout North Africa, the Middle East, and Southern and Eastern Europe. These parasitic weeds form an haustorium at the tip of their radicles to infect the crop upon detection of the host-derived haustorium-inducing factors. Until now, the haustorial induction in the broomrapes remains less studied than in other parasitic plant species. Known haustorium-inducing factors active in hemiparasites, such as *Striga* and *Triphysaria* species, were reported to be inefficient for the induction of haustoria in broomrape radicles. In this work, the haustorium-inducing activity of *p*-benzoquinone and 2,6-dimethoxy-*p*-benzoquinone (BQ and DMBQ) on radicles of three different broomrapes, namely *Orobanche cumana*, *Orobanche minor* and *Phelipanche ramosa*, is reported. Additional allelopathic effects of benzoquinones on radicle growth and radicle necrosis were studied. The results of this work suggest that benzoquinones play a role in the induction of haustorium in broomrapes. Although dependent on the broomrape species assayed and the concentration of quinones used in the test, the activity of BQ appeared to be stronger than that of DMBQ. The redox property represented by *p*-benzoquinone, which operates in several physiological processes of plants, insects and animals, is invoked to explain this different activity. This work confirms the usefulness of benzoquinones as haustorium-inducing factors for holoparasitic plant research. The findings of this work could facilitate future studies in the infection process, such as host-plant recognition and haustorial formation.

## 1. Introduction

Approximately 1% of all angiosperms distributed among 28 dicotyledonous families are plant parasites. Some are facultative parasites, capable of living autotrophically, but adopting a parasitic lifestyle when a susceptible host is nearby. In comparison, obligate parasitic plants require host infection shortly after germination. Parasitic plants differ in their ability to photosynthesize, being grouped either as photosynthetically competent hemiparasites or achlorophyllous holoparasites. They can also be grouped by the host plant organ they infect, either as root parasitic plants that infect host roots or shoot parasitic plants that infect host stems [1,2,3,4,5,6,7]. Parasitic plants have evolved the parasitic lifestyle independently at least 12 times, and the key feature of this evolution is a unique multicellular organ called the haustorium [4].

The haustorium has successive functions of host attachment, invasion and connection to the host’s vascular system. The first developmental stage for the formation of a mature haustorium in root parasitic plants, is the initiation of a pre-attached haustorium by host-derived signals [7,8,9]. In root hemiparasitic plants, the development of a pre-attached haustorium is visually observed with a cessation of parasitic root growth and a swelling of the root tip with an outgrowth of epidermal cells that form the haustorial hairs with the activity of host adherence [8]. Similarly, in the root holoparasitic *Orobanche* and *Phelipanche* species, the pre-attached haustorium is observed by a cessation of the radicle growth with a swelling of radicle tip, but in the broomrape case, a layer of short cell extensions called papillae is formed, which also have adhesive functions [10,11]. Pre-attached haustoria in root hemiparasites is induced by haustorium-inducing factors (HIFs) released from the host roots. Phenolic acids and flavonoids were reported to induce haustoria in hemiparasites [12,13,14,15]. The haustorial induction in radicles of broomrapes was considered independent from exogenous factors [4,10], and while it has recently been demonstrated that broomrape radicles respond to chemical induction with haustorium formation [11,16,17,18], their HIFs remain largely unknown today.

*p*-Benzoquinones or 1,4-benzoquinones are a subgroup of natural quinones, well known as metabolites produced by higher plants, fungi, bacteria and animals [19,20]. They are involved in important biological processes as bioenergetic transport, oxidative phosphorylation and electron transport, which confer potent antioxidant, anti-inflammatory and anticancer activities. Many studies report the mode of action of *p*-benzoquinones [21], and their biological activities prompted an investigation into their therapeutic application in medicine. Their therapeutic potential depends on the different substitution patterns that regulate a varied range of different cellular pathways and different selective activities [22]. Contrary to medical applications, their potential in agriculture has been poorly explored. Humic acids can be obtained by the alkaline transformation of *p*-benzoquinones for a potential application in agriculture [23]. *p*-Benzoquinone (BQ) and 2,6-dimethoxy-*p*-benzoquinone (DMBQ) are by-products in pretreated lignocellulosic biomass, which inhibit microorganism fermentation [24]. BQ has been used to evaluate the effects of race and age in cattle on the redox condition of their blood plasma [25]. 

DMBQ was identified from the roots of sorghum [13,26], a host crop for the parasitic weed *Striga.* Haustorium-inducing activity was identified in DMBQ from the hemiparasitic plants *Striga*, *Triphysaria, Agalinis* and *Phtheirospermum* [13,14,15,27,28,29,30]. During crop–parasitic plant interactions, BQ derivatives are generated in host roots from monolignol by host peroxidase at a close distance to the root tip of hemiparasites [27]. Contrary to hemiparasites, the DMBQ haustorial-inducing activity was not reported in broomrapes [4,16,17,18]. In this study, we screened BQ and DMBQ for their ability to induce haustorium development in the radicles of three broomrape species, *Orobanche minor, Orobanche cumana* and *Phelipanche ramosa*. Additional allelopathic effects of benzoquinones on radicle growth and necrosis are also reported. Our research identified the activity of benzoquinones as HIFs for broomrape species for the first time. 

## 2. Results and Discussion

**Figure 1:**
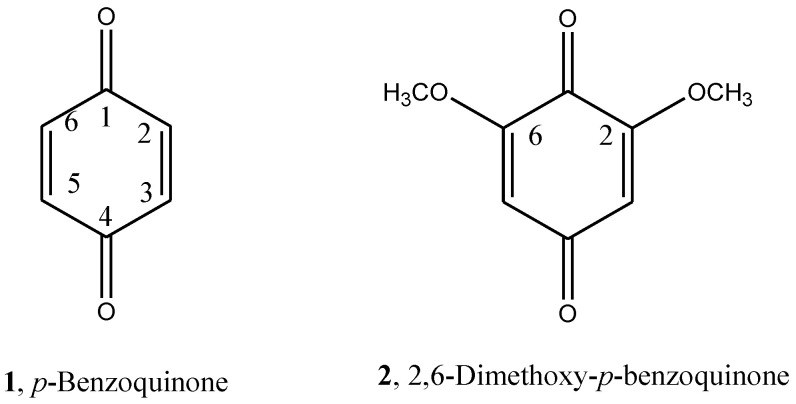
Structures of BQ and DMBQ (**1** and **2**).

We used an in vitro system to assay the effects of benzoquinone structure (BQ and DMBQ, **1** and **2** shown in Figure 1), concentration (0.01, 0.05, 0.1, 0.2, 0.4, 0.6, 0.8, and 1 mM) and broomrape species (*Orobanche cumana, Orobanche minor, Phelipanche ramosa*) on broomrape radicle growth, radicle necrosis and haustorium induction.

### 2.1. Radicle Growth

**Figure 2:**
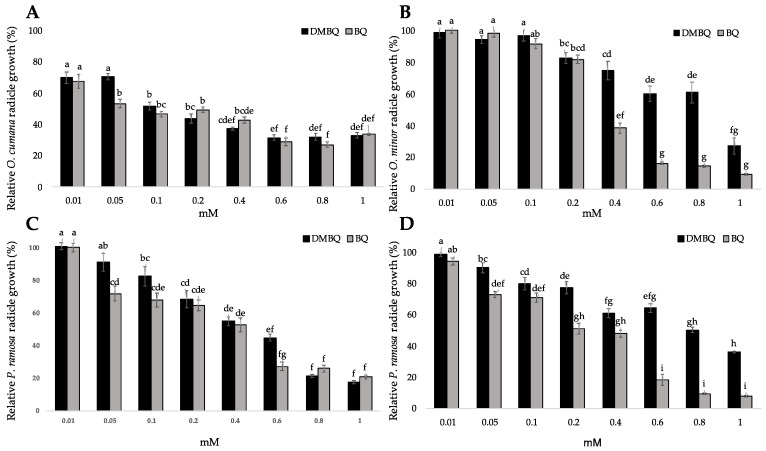
The effects of DMBQ and BQ on the growth of broomrape radicles. (**A**) *O. cumana*; (**B**) *O. minor*; (**C**) *P. ramosa* population 1; (**D**) *P.* *ramosa* population 2. For each broomrape species, treatments with different letters are significantly different according to the Tukey test (*p* = 0.05). Error bars represent the standard error of the mean.

When broomrape radicles were exposed to benzoquinones, there was a significant reduction in radicle growth, relative to control radicles. Significant effects on radicle growth were observed for benzoquinone structure, benzoquinone concentration and broomrape species (ANOVA, *p* < 0.001, *p* < 0.001 and *p* < 0.001 respectively; Figure 2). Both benzoquinones were active in inhibiting the radicle growth of all broomrape species at the concentrations tested in a range of 0.1–1 mM. The effect of BQ on radicle growth was stronger than the effect of DMBQ. The BQ half-maximal growth inhibitory concentration (IC_50_) were 0.19, 0.46, 0.46 and 0.35 mM, respectively for *O. cumana, O. minor, P. ramosa* population 1 and *P. ramosa* population 2. While IC_50_ for DMBQ was 0.29, 0.78, 0.52 and 0.76 mM, respectively for *O. cumana, O. minor, P. ramosa* population 1 and *P. ramosa* population 2. At 0.05 mM, only BQ significantly inhibited radicle growth in all species except for *O. minor*. The *P. ramosa* population 1 was more sensitive to both benzoquinones, achieving an average radicle growth in treatments with BQ and DMBQ at 1 mM of 21.0% ± 1.1 and 17.7% ± 0.9, respectively, compared to the radicles control. The radicles of *O. cumana* were less sensitive, showing an average growth of 33.7% ± 0.7 and 32.9% ± 1.8 in treatments with BQ and DMBQ at 1 mM, respectively, compared to the control. The perception of haustorium-inducing factors promotes cessation of parasite root growth [31]. The root elongation resumes in facultative root parasitic plants, which characteristically form lateral haustorium, but growth remains repressed in obligate root parasitic plants, which characteristically form terminal haustorium at the tip of their radicles [4,31]. 

### 2.2. Radicle Necrosis

**Figure 3:**
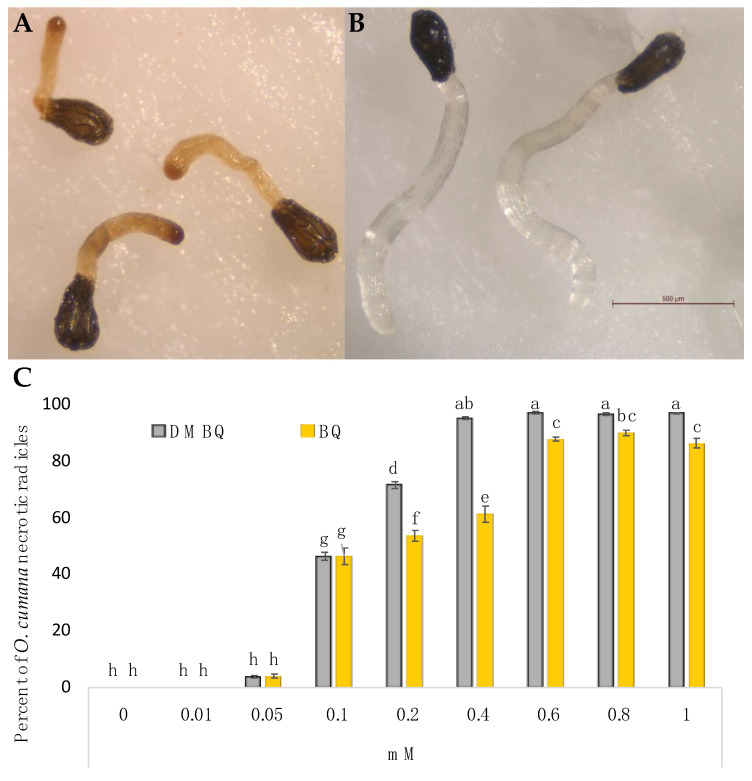
Necrosis developed by *Orobanche cumana* radicles treated with benzoquinones. (**A**) *O. cumana* radicles treated with DMBQ applied at 0.8 mM; (**B**) *O. cumana* control radicles; (**C**) Dosage response to DMBQ and BQ. Treatments with different letters are significantly different according to the Tukey test (*p* = 0.05).

Significant effects on radicle necrosis were observed for benzoquinone structure, concentration and broomrape species (ANOVA, *p* < 0.001, *p* < 0.001 and *p* < 0.001 respectively). DMBQ was reported to be toxic to *Triphysaria* roots at concentrations of 100 μM or higher [32], while DMBQ was toxic to *Striga* radicles at 50 μM or higher concentrations [27,33]. In this work, toxicity was not observed in radicles of *O. minor*, or in radicles of *P. ramosa* at any concentrations tested, including those concentrations previously reported as toxic for *Striga* and *Triphysaria* roots. Contrary to *O. minor* and *P. ramosa,* radicles of *O. cumana* responded with necrosis to the benzoquinone treatments. Both benzoquinones tested induced significant *O. cumana* necrosis at a concentration range of 0.1 to 1 mM (Figure 3). The concentrations required to induce half-maximal necrosis in *O. cumana* radicles (N_50_) were 0.35 and 0.25 mM for BQ and DMBQ, respectively. At concentrations of 0.2 and 0.4 mM, the necrosis-inducing effect on *O. cumana* was significantly higher for DMBQ than for BQ. The phytotoxic effect of *p*-quinones was suggested to have an application in integrated pest management. Among phytotoxic *p*-quinones there are 2- and 3-hydroxyjuglone, botrytone, regiolone, *cis*- and *trans*-2,4,8-trihydronaphthalenones, isolated from *Botrytis fabae* [34]; scytalone, regiolone, *cis*-4-hydroxyscytalone, 1,3,8-trihydroxynaphthalene, 3,4,8-trihydroxytetralone, 2,4,8-trihydroxytetralone, and flaviolin isolated from *Phaeoacremonium minimum*, *Phaeomoniella chlamydospora*, and *Neofusicoccum parvum* [35]; and diploquinones A and B isolated from *Diplodia mutila* [36]. 

### 2.3. Induction of Haustorium

**Figure 4:**
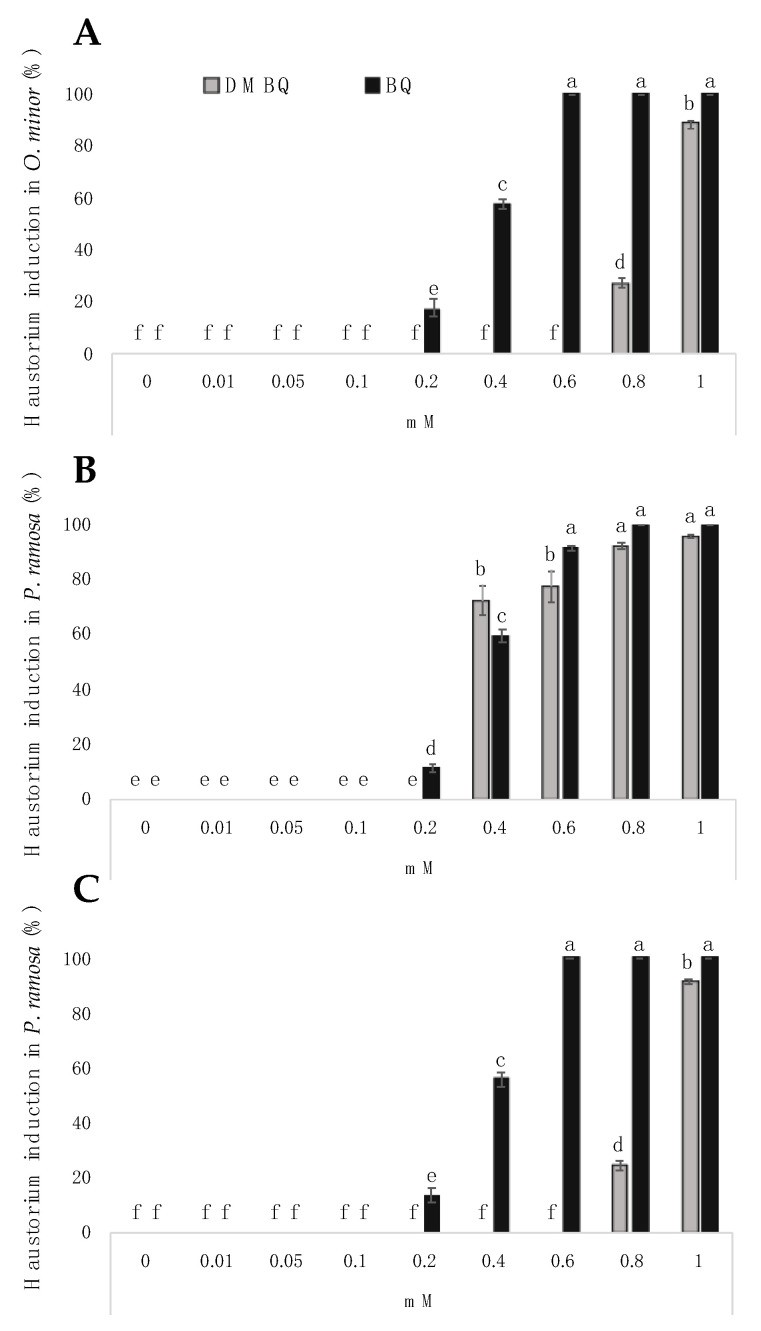
Haustorium-inducing effects DMBQ and BQ on broomrape radicles. (**A**) *O. minor*; (**B**) *P.ramosa* population 1; (**C**) *P. ramosa* population 2. Haustorium was not induced in *O. cumana* radicles by any of the compounds tested. For each broomrape species, treatments with different letters are significantly different according to the Tukey test (*p* = 0.05).

Significant effects on haustorium induction were observed for benzoquinone structure, concentration and broomrape species (ANOVA, *p* < 0.001, *p* < 0.001 and *p* < 0.001 respectively, Figure 4). DMBQ was reported to be active in inducing *Triphysaria* haustorium between 1 and 30 μM concentrations [32]. In *Striga* species, the active range spans from 0.05 to 10 μM [27,33]. Unlike *Striga* spp. and *Triphysaria* spp., the broomrape species were reported to not respond to DMBQ with haustorium initiation [4,10,16,17,18], but the activity of DMBQ was tested in broomrape radicles only at 10 μM [18]. In our work, both benzoquinones tested induced broomrape haustoria. The haustorium-inducing effect was active on *O. minor* and in both populations of *P. ramosa* tested, but not in *O. cumana*. The BQ concentrations required to induce half-maximal haustorium in broomrape radicles (H_50_) were 0.42 and 0.43 mM, respectively, for *O. minor* and *P. ramosa* population 2, while DMBQ H_50_ was 0.92 mM for both of these broomrape species. At concentrations tested in a range from 0.2 to 0.8 mM, the haustorium-inducing activity of BQ was higher than the activity of DMBQ in radicles of *O. minor* and *P. ramosa* population 2. Both DMBQ and BQ were similarly active in radicles of *P. ramosa* population 1 with a respective H_50_ of 0.46 and 0.43 mM. The results presented in this manuscript suggest a common mechanism for benzoquinone perception in broomrape species, *Striga* and *Triphysaria,* consistent with their common origins [4]. 

DMBQ is released from the host cell wall by oxidation upon host peroxidase activity triggered by hydrogen peroxide production at the tip of the parasitic radicle [27]. According to this model, the events of HIFs release by the host and subsequent haustorium development by the parasite occur when the parasite is close to the host root [27]. The HIFs concentration in the rhizosphere will follow a concentration gradient inversely related with the host distance. The differences in required concentration of the HIFs between *Striga hermonthica* and broomrapes may partly be related to the fact that broomrape radicle is shorter and elongates more slowly than *Striga* radicle. As the broomrape haustorium is terminal, anticipated induction of haustorium before reaching the host root would be lethal for the broomrape seedling, and therefore a requirement of higher concentration of HIFs may be favorable for a successful broomrape parasitism to avoid a suicidal induction of haustorium. Despite recent research intensification, the mechanisms leading to haustorium formation in *Orobanche* and *Phelipanche* species remain largely unknown. Recent transcriptomic studies identified parasitism genes expressed during haustorium induction in obligated parasitic weeds *S. hermonthica* and *Phelipanche aegyptiaca* [37]. During the work of Yang et al. [37], DMBQ was used to induce the haustorium in *S. hermonthica* radicles, while host roots were used to induce the haustorium of *P. aegyptiaca*; however, at the time of Yang et al.’s [37] studies, the haustorial induction activity of benzoquinones on broomrape was unknown. The present work provides valuable information for future research.

The process of haustorium development was reported to be more sensitive to cellular redox states than root growth [38], but in broomrape we found that the reduction in radicle growth was induced at lower benzoquinone concentrations than those concentrations active for haustorium induction. It has been debated whether the growth cessation is necessary for haustorium functioning, or if it is the consequence of a reduction in resources required for root growth [31]. Endogenous auxin levels increase after DMBQ treatment [39]. Root elongation is inhibited by auxin-stimulated ethylene production [40]. One potential approach for controlling obligated parasitic weeds in the field would be to breed for HIFs overexcreting crop varieties that repress the growth of the parasitic radicle prematurely before host contact. Another strategy for controlling broomrape in the field would be to inhibit the haustorium development, either by breeding host crop varieties with reduced haustorium-inducing activity [41] or by intercropping susceptible crops with allelopathic crops with inhibitory activity on haustorium development [42].

### 2.4. Species-Specific Activity

**Figure 5:**
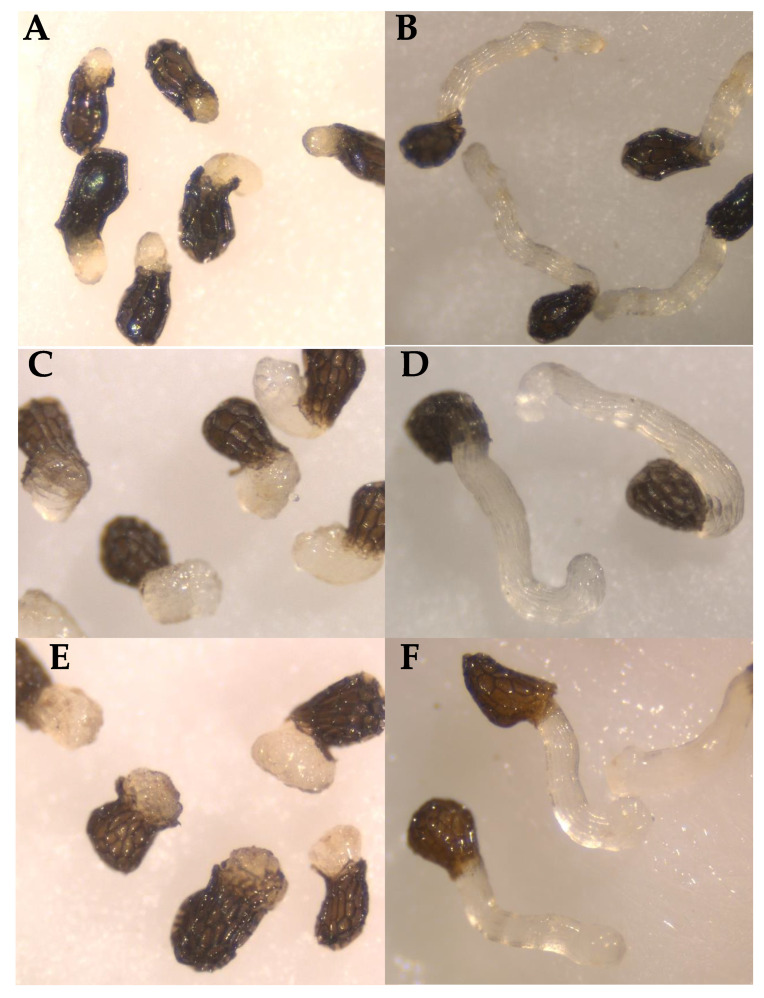
Haustorium-inducing effects of BQ on broomrape radicles. (**A**) *O. minor*; (**C**) *P. ramosa* population 1; (**E**) *P. ramosa* population 2. Growth of control broomrape radicles (**B**) *O. minor*; (**D**) *P. ramosa* population 1; (**F**) *P. ramosa* population 2.

The interaction of broomrape species × benzoquinone structure, the interaction broomrape species × benzoquinone concentration and the triple interaction of broomrape species × benzoquinone structure × benzoquinone concentration had significant effects on radicle growth (ANOVA, *p* < 0.001, *p* < 0.001 and *p* < 0.001 respectively), radicle necrosis (ANOVA, *p* < 0.001; *p* < 0.001 and *p* < 0.001 respectively) and haustorium induction (ANOVA, *p* < 0.001, *p* < 0.001 and *p* < 0.001 respectively). The haustorium-inducing effect of BQ and DMBQ was active on *O. minor* and on both populations of *P. ramosa* tested, but not on *O. cumana* radicles. *O. cumana*, is a specific parasite of sunflower, which was described to have different chemical requirements to detect the host when compared with the rest of broomrape weed species [43]. The growth inhibition activity of BQ and DMBQ was active in all broomrape species, including *O. cumana*. Instead of haustorium formation, *O. cumana* radicles responded to BQ and DMBQ detection with radicle necrosis. A recent publication by Jiang et al. [44] described the effect of DMBQ at 0.2 mM on radicles of a Chinese population of *O. cumana* race G. The phenotype observed by Jiang et al. [44] was similar to the phenotype we observed in radicles of our Turkish population of *O. cumana* (Figure 3A), and was different from the haustorial phenotype we observed in radicles of *Orobanche minor* (Figure 5A) and *P. ramosa* (Figure 5 C,E).

The activity of DMBQ was stronger in radicles of *P. ramosa* population 1 than in radicles of *O. minor* and *P. ramosa* population 2. The *P. ramosa* population 1 collected in winter oilseed rape was more sensitive to both BQ and DMBQ for haustorium induction and radicle growth cessation than *O. minor* and *P. ramosa* population 2 collected in hemp, which were mostly sensitive to BQ. Differences in sensitivity for the detection of germination inductors have previously been reported between *P. ramosa* populations [45]. Huet et al. [45] reported that seeds collected from *P. ramosa* plants infecting winter oilseed rape were more sensitive to germination induction factors than those collected in hemp. Species specificities have previously been described for HIF recognition [14]. Polymerization of monolignols results in the *p*-hydroxyphenyl- (H), guaiacyl- (G), and syringil- (S) derivatives, which showed nil, one, and two methoxy groups bonded to the aromatic rings, respectively. S-type compounds induce haustoria in both *S. hermonthica* and *P. japonicum*. In contrast, G-type compounds have a high capacity for inducing haustoria in *S. hermonthica*, but not in *P. japonicum*. H-type compounds do not induce haustorium formation [14,18].

**Figure 6:**
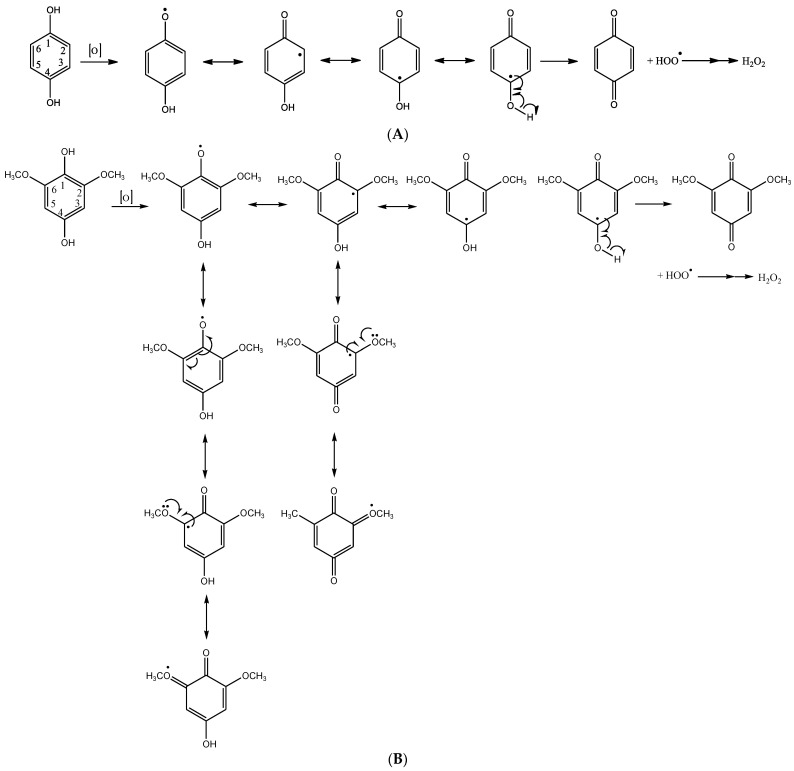
(**A**) The equilibrium between the hydroquinone and quinone forms of BQ. (**B**)The equilibrium between the hydroquinone and quinone forms of DMBQ.

Quinone/hydroquinone structures serve as cofactors in many metabolic pathways, playing critical roles in oxidation/reduction processes. Many *p*-benzoquinone derivatives have been identified in nature, such as vitamin K_1_ (phylloquinone) of vegetable origin, which intervenes in the blood coagulation processes; the yellow pigment contained in the leaves of the tropical bush known as henna (*Lawsonia inermis*) and the quinone complex known as coenzyme Q essential in oxidative phosphorylation. Many insects and arthropods use *p*-benzoquinones for defense [46]. The fundamental reaction that regulates this equilibrium is shown in Figure 6A. The presence of the substituents, their position and nature can differently affect the redox equilibrium of BQ, as reported in Figure 6B for DMBQ.

The presence of methoxy groups at C-2 and C-6 in DMBQ further stabilizes the radical intermediate favoring the shift of equilibrium towards the quinonic form. Although the effects of both BQ and DMBQ are also parasitic plant species-dependent, the equilibrium reported in Figure 6A,B could be invoked to explain the stronger effect on the inhibition of radicle growth, induction of necrosis and haustorium formation of BQ in respect to DMBQ.

The *p*-benzoquinones can have an opposite role when produced by fungi or by plants. In fact, juglone and plumbagin, derivatives of BQ occurring in plants, are able to generate reactive oxygen species (ROS), which play an important role in the processes of programmed cell death during plant defense. These are in agreement with the activity observed in BQ and DMBQ. Each different physiological process could be affected differently by the *p*-benzoquinone redox property and the plant species tested [47,48]. Conversely, pathogenic fungi produce *p*-quinones to inhibit the ROS produced as a defense by plants in the infected tissues [49]. This mechanism was probably used by the diploquinones A and B, two differently tetrasubstituted BQ produced from *D. mutila* infecting grapevine, as above cited. Their different phytotoxicity could be related to their different substitution pattern of both rings. In fact, the presence of an *ortho*-diphenol moiety in diploquinone A facilitates its oxidation, probably enhancing its interaction with the oxidative systems of plant cells [36]. This mode of action could also explain the phytotoxicity of foeniculoxin, a geranylhydroquinone isolated from *Phomopsis foeniculi*, the causal agent of fennel stem necrosis [50]. A similar mechanism of action could explain the haustorium-inducing activity of sphaeropsidones in radicles of the parasitic weeds *Striga* and *Orobanche* [11].

## 3. Materials and Methods

### 3.1. Plant Materials, Reagents 

*p*-Benzoquinone (BQ) and 2,6-dimethoxy-*p*-benzoquinone (DMBQ) were obtained from Sigma-Aldrich (cat. no. B10358 and 428566, respectively). A set of four broomrape seed accessions from the parasitic seed collection of the Institute for Sustainable Agriculture-Spanish National Research Council (IAS-CSIC) was used to identify the allelopathic effects of benzoquinones on radicle growth, necrosis and haustorium induction. According to their morphology, host range and response to germination stimulants [43,45,51,52], the four broomrape accessions were classified in two *Orobanche* species: *Orobanche cumana*, race G collected on sunflower in Turkey and *Orobanche minor* population collected on red clover in France, and two French populations of *Phelipanche ramosa*, population 1 collected on winter oilseed rape and population 2 collected on hemp.

### 3.2. Screening of Allelopathic Activity

In vitro bioassays were carried out according to previous protocols [11,53,54]. The seeds of three broomrape species indicated above were surface sterilized by immersion in 0.5% (*w*/*v*) NaOCl and 0.02% (*v*/*v*) Tween 20, for 5 min, rinsed thoroughly with sterile distilled water, and dried in a laminar air flow cabinet. Broomrape germination is induced in the laboratory through a two-step process, a warm stratification called conditioning followed by a chemical induction by the synthetic strigolactone GR24 [55]. To achieve seed conditioning, approximately 100 seeds of each broomrape species were placed separately in 9 mm diameter glass fiber filter paper disks (GFFP; Whatman International Ltd., Maidstone, UK) moistened with 50 μL of sterile distilled water and placed inside Petri dishes in incubators at 23 °C in the dark for 10 days to allow seed conditioning. GFFP disks containing conditioned broomrape seeds were transferred onto a sterile sheet of filter paper to remove the excess of water, and then transferred to new 10 cm sterile Petri dishes. Fresh stocks of BQ and DMBQ were prepared right before the seed treatment in sterile distilled water to avoid toxic effects of organic solvents on broomrape radicles. Triplicate aliquots of 100 μL of BQ and DMBQ at 0.00 (control), 0.01, 0.05, 0.1, 0.2, 0.4, 0.6, 0.8, and 1 mM, individually mixed with the synthetic germination stimulant GR24 10^−6^ M, were applied to GFFP discs directly on top of the broomrape conditioned seeds. Petri dishes were sealed with parafilm and incubated in the dark at 23 °C for 7 days.

### 3.3. Calculations and Statistical Analysis

Using a stereoscopic microscope (Leica S9i, Leica Microsystems GmbH, Wetzlar, Germany) the number of radicles that developed haustorium and the radicle length were measured. Note was also taken on each seedling of whether radicle had developed necrosis. The percentage of seedlings that developed haustorium and the percentage of seedlings that developed a necrotic radicle was calculated in each triplicated GFFP disk for each treatment. For the characteristic of radicle growth, the value used was the average of 10 randomly selected radicles [56]. The percentage of radicle growth inhibition of each treatment was then calculated relative to the average radicle growth of control treatment. Percentages were transformed to arcsine square roots (transformed value = 180/Π × arcsine [√(%/100)]) to normalize data and stabilize variances throughout the data range, and subjected to an analysis of variance (ANOVA) using SPSS software. The significance of mean differences between treatments was evaluated by the Tukey test. The null hypothesis was rejected at the level of 0.05.

## 4. Conclusions

Obligate parasitic weeds are important agricultural pests. Despite recent research intensification, the mechanisms leading to haustorium induction in *Orobanche* and *Phelipanche* species remain largely unknown. This study characterized the activity of *p*-benzoquinone and 2,6-dimethoxy-*p*-benzoquinone in radicles of *O. cumana*, *O. minor* and *P. ramosa*. The results suggest that pre-attached haustoria in broomrape is inducible by benzoquinones. However, the activity is dependent on benzoquinone structure, concentration and broomrape species. These findings provide a new perspective and facilitate future research on broomrape parasitism. They also open new research possibilities in crop protection through the design of strategies that disrupt crop–parasite communication, or through breeding crops for novel mechanisms of broomrape resistance based in roots with a reduced production of HIFs that could lead to reduced parasitism.

## Data Availability

The data presented in this study are available on request from the corresponding author.
